# Make it bloom! CONSTANS contributes to day neutrality in rose

**DOI:** 10.1093/jxb/eraa270

**Published:** 2020-07-06

**Authors:** Béatrice Denoyes, Amèlia Gaston, Christophe Rothan

**Affiliations:** INRAE and University of Bordeaux, UMR 1332 Biologie du Fruit et Pathologie, Villenave d’Ornon, France

**Keywords:** CONSTANS, day neutrality, continual flowering, domestication, diversification, rose, Rosaceae, strawberry

## Abstract

This article comments on:

**Lu J, Sun J, Jiang A, Bai M, Fan C, Liu J, Ning G, Wang C**. 2020. Alternate expression of CONSTANS-LIKE 4 in short days and CONSTANS in long days facilitates day-neutral response in *Rosa chinensis*. Journal of Experimental Botany **71**, 4057–4068

**Day neutrality is an important agronomical trait in crop species such as rose (*Rosa chinensis*), a major cut-flower crop, allowing continuous flowering (CF) whatever the daylength. In Lu *et al.* (2020), the authors highlighted the role of the CONSTANS-LIKE 4 (RcCOL4) and CONSTANS (RcCO) proteins in the regulation of the florigen FLOWERING LOCUS T (*RcFT*) expression under long-day (LD) and short-day (SD) conditions, respectively. They further showed that RcCO binds to the CORE motif of the *RcFT* promoter and proposed a model in which RcCOL4 interacts with RcCO to enhance *RcFT* expression. Altogether, their results showed that RcCO and RcCOL4 contribute to day neutrality in CF rose.**

## Day neutrality is a major domestication and diversification trait

Flowering plants are widely distributed on earth and must therefore cope with diverse environmental conditions, ranging from the highly contrasted temperature and light conditions across days and seasons in high latitudes and altitudes (Gaston *et al.*, 2020) to the much greater environmental stability in equatorial areas. Plant species have adapted the timing of their flowering to these specific environments, in order to achieve their reproductive cycle in favourable conditions and produce seeds in due time. Daylength is one of the most prominent environmental cues controlling seasonal flowering. Flowering is induced by increased daylight in long-day (LD) plants, by reduced daylight in short-day (SD) plants, but is insensitive to the photoperiod in day-neutral (DN) plants.

The induction of flowering is controlled by the complex balance between florigen and antiflorigen, and by their interactions with the photoperiod and day/night alternation (circadian cycle) sensing and signalling pathways (Matsoukas, 2015). The CENTRORADIALIS/TERMINAL FLOWER 1/SELF-PRUNING (CETS) family members, including FLOWERING LOCUS T (FT), TERMINAL FLOWER1 (TFL1), and their related proteins, have evolved to play prominent roles as florigen or antiflorigen in many plant species. The transcription factor CONSTANS (CO) integrates the light and circadian clock signals and, by complex mechanisms involving CO protein transcription and protein stability in response to light, activates or represses the FT florigen (Box 1), thus playing a central role in the regulation of flowering (Song *et al.*, 2015). How variations in the photoperiodic pathway genes have evolved in natural conditions and upon domestication and diversification to control plant production period and yield in SD and LD conditions, for example through continuous flowering (CF), is a vivid field of research in many crop species, including the *Rosaceae* species strawberry and rose (Iwata *et al.*, 2012; Tenreira *et al.*, 2017). Lu *et al.* (2020) pinpoint the contribution of the CO proteins to day neutrality in rose and provide further insights into how the interactions between the various rose COs regulate flowering in response to variations in photoperiod.

Box 1. **Photoperiodic pathway genes targeted during domestication for loss of photoperiod sensitivity in crop species.**

In a simplified view of the photoperiodic flowering pathway of the dicotyledonous model *A. thaliana*, light duration and quality are perceived by photoreceptors (phytochromes PHYA and PHYB) and the circadian clock. The circadian clock regulates the protein complex formed by GIGANTEA (GI) and FLAVIN BINDING, KELCH REPEAT, F-BOX 1 (FKF1). This GI–FKF1 complex stabilizes CONSTANS (CO) through degradation of the CO repressor CYCLING DOF FACTOR 1 (CDF1). CO plays a central role in the photoperiodic pathway by integrating the light signal and, in turn, by promoting the expression of the florigen FLOWERING LOCUS T (FT), a member of the CETS family. FT protein migrates through phloem into the shoot apical meristem (SAM) where it competes with the floral repressor TERMINAL FLOWER 1 (TFL1), another member of the CETS family, and promotes flowering through the activation of the floral meristem identify genes (Matsoukas, 2015).

During the domestication and subsequent diversification, mutations in photoperiodic flowering pathway genes allowed crop species to overcome their sensitivity to photoperiod, spread outside their centre of origin, and increase their yield. Allelic variations underlying these traits in various crops are represented by a triangle for monocots, and by a square (Asterid clade) or a circle (Rosid clade) for dicots. Only allelic variations in genes homologous to *A. thaliana* photoperiodic and flowering pathway genes are indicated for the various species. The photoperiodic pathway from monocots includes specific genes with no clear homology to *A. thaliana* [e.g. *Ghd7*, *Ehd1*, and *Ppd-H1* (Olsen and Wendel, 2013; Blackman, 2017] that are therefore not represented. Positive and negative regulatory connections are indicated by arrows and bar-ended lines, respectively.

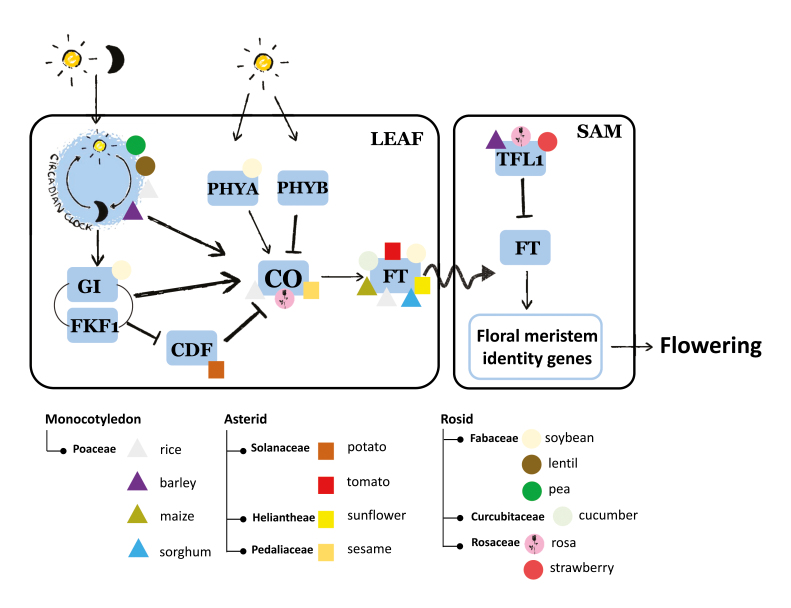

## CONSTANS studies shed light on the mechanisms underlying the continuous flowering trait in rose

Lu *et al.* (2020) aimed at finding new regulators of the season-independent CF trait in rose. Day neutrality is a target trait for modern rose breeding that was first introgressed from CF Chinese rose (*Rosa chinensis*) into European varieties by 18th century breeders (Iwata *et al.*, 2012). Because of the conserved role of the CO/FT pathway in *Arabidopsis thaliana*, rice, and other crops (Box 1), Lu *et al.* (2020) focused their investigation on the CO and CO-like proteins, which are photoperiod-responsive proteins containing the B-box (BBX) zinc finger domains found in numerous transcription factors (Bai *et al.*, 2019). Among the 18 non-redundant BBX genes they identified, they focus their study on the three closest to Arabidopsis *AtCO* (*AtBBX1*)-like proteins with a highly conserved double BBX domain in the N-terminus and a CCT domain in the C-terminus. They were named *RcCO* (*RcBBX1*), *RcCOL4* (*RcBBX5*), and *RcCOL5* (*RcBBX6*).

*CO* photoperiod-dependent expression is highly reliant on plant species. For example, Arabidopsis *AtCO* is highly expressed in LDs while its rice orthologue *HEADING-DATE1* (*Hd1*) is highly expressed in SDs (Olsen and Wendel, 2013). Lu *et al.* (2020) therefore analysed the circadian expression of *RcCO*, *RcCOL4*, and *RcCOL5* in the CF variety (*tfl1* mutant) ‘Old Blush’, in both LD and SD conditions. All three genes were regulated by the circadian cycle. The photoperiodic expression patterns of *RcCO* and *RcCOL4* were, however, opposite, with the *RcCO* transcript level being higher in LDs and that of *RcCOL4* higher in SDs. *RcCOL5* was insensitive to photoperiod. Next, Lu *et al.* (2020) showed that these photoperiodic patterns were conserved in the *Rosa* genus by analysing both season-dependent once flowering (OF) and season-independent CF genotypes from different *Rosa* species. Specifically, *RcCO* and *RcCOL4* consistently displayed opposite responses in CF roses, while *RcCOL5* expression was similar in either SDs or LDs in both CF and OF genotypes.

Because the stable genetic transformation of rose is a long process, Lu *et al.* (2020) then used an established virus-induced gene silencing (VIGS) technique to show that *RcCO*- and *RcCOL*-silenced plants (*tfl1* mutant) flowered later whatever the gene considered, thus indicating that *RcCO*, *RcCOL4*, and *RcCOL5* are all three activators of flowering in rose. In addition, the flowering delay was different in LDs and SDs in *RcCO-* and *RcCOL4*-silenced plants, respectively, thus suggesting a different role for these two genes in the DN response. This hypothesis was further supported by the observation that the transcript level of *RcFT*, the downstream florigen target of *RcCO*, was higher in SDs than in LDs in *RcCO*-silenced plants; the opposite was true for *RcCOL4*-silenced plants. Thus, the successive expression of *RcCO* in LDs and of *RcCOL4* in SDs would allow the expression of *RcFT* independently of the photoperiod, thereby facilitating the DN response of roses.

In *A. thaliana*, AtCO regulates *AtFT* expression by binding to the CORE motif in the *FT* promoter (Song *et al.*, 2015). Using an efficient rose leaf transient expression system previously set up by the team (Lu *et al.*, 2017), Lu *et al.* (2020) showed that both RcCO and RcCOL4 can bind to the *RcFT* promoter and activate its expression. By combining the transient expression experiments with the VIGS of either *RcCO* or *RcCOL4*, they further observed that when *RcCO* was silenced the RcCOL4 protein could not induce *RcFT* expression, while when *RcCOL4* was silenced *RcFT* was expressed. This result was further supported by EMSAs showing that RcCO but not RcCOL4 could bind to the CORE motif present in the *RcFT* promoter. It is noteworthy that the binding activity of RcCO to the CORE motif was enhanced when RcCO was combined with RcCOL4. This probable interaction was next investigated through mutagenesis experiments in which Box1 or Box2 from RcCOL4 were mutated. As a result, the RcCOL4–RcCO protein–protein interaction was no longer observed after Box1 mutagenesis, while Box2 mutagenesis had no effect, thus highlighting the role of Box1 in RcCO–RcCOL4 interaction.

Lu *et al.* (2020) proposed a schematic model of the regulation of the flowering time by RcCOL4–RcCO in *R. chinensis* according to the daylength. Under LD conditions, RcCO promotes flowering via direct binding to the *RcFT* promoter to activate its expression, while under SD conditions RcCO is down-regulated and RcCOL4 accelerates flowering via physically interacting with RcCO to enhance its binding to *RcFT*. Consequently, the *R. chinensis* CF ‘Old Blush’ variety in which the floral repressor TFL1 is absent could flower under both LDs and SDs.

## Loss of photoperiod sensitivity in the *Rosaceae* family and in other crop species

A key domestication trait in many crop species was the acquisition of day neutrality (Soyk *et al.*, 2017). Wild ancestors of cultivated crops are well adapted to the environmental conditions encountered in their centre of origin. Spread of domesticated species required their adaptation to novel conditions well beyond their restricted original environment, for example high latitudes. A major target of crop domestication and improvement is yield (grain, tubers, fruit, flowers, and so on) which, in many cultivated species, can be improved through the modification of plant architecture, such as for increasing the number of flowers (Tenreira *et al.*, 2017; Gaston *et al.*, 2020) and by extending the production period via the acquisition of daylength insensitivity and therefore loss of seasonality (Blackman, 2017). This goal has been achieved in a wide range of crop species (Box 1).

The molecular mechanisms underlying light and circadian clock regulation of photoperiodic responses and the integration of the photoperiodic pathway into the flowering regulatory network have been recently deciphered in the model dicot Arabidopsis (Box 1). Core components of these pathways (GI, CDFs, CO, and FT) are shared with the monocots, which have additionally evolved specific functions. To date, allelic variations underlying the acquisition of day neutrality have been identified in almost all the major components of the photoperiodic flowering pathway. Among them, the CETS family members FT and TFL1, whose balance controls the induction of flowering, were instrumental in the adaptation of crops to their new environment and to yield requirements. Examples include not only the dicots such as the tomato (*Solanaceae*; Soyk *et al.*, 2017) and the strawberry and rose (*Rosaceae*; Iwata *et al.*, 2012) but also the monocots rice, maize, barley, and sorghum (Takahashi *et al.*, 2009; Comadran *et al.*, 2012; Blackman, 2017; Guo *et al.*, 2018). An additional key target of the photoperiodic pathway is CO, as revealed in sesame and rice (Takahashi *et al.*, 2009; Olsen and Wendel, 2013; Zhou *et al.*, 2018; Lu *et al.*, 2020). CO plays a central role by integrating the upstream light and clock signals and by translating the information into floral induction or repression via the floral activator FT. The light and circadian clock perception and signalling machinery upstream of CO is also involved in the acquisition of photoperiod insensitivity in pea, lentil, and soybean (Olsen and Wendel, 2013; Blackman, 2017).

Lu *et al.* (2020) further demonstrated that two of the rose CO paralogues interact and participate in the establishment of day neutrality in rose. There are indications that CO may control flowering time in other *Rosaceae* species including woodland strawberry (Kurokura *et al.*, 2017) and loquat (Zhang *et al.*, 2019). The *Rosaceae* is a major plant family including crop species as diverse as roses but also the small fruit species strawberries, raspberries, blueberries, etc., the fruit tree species apple, peach, cherry, apricot, almond, etc., and the less widespread loquat. Several lines of evidences point to the possible transfer of the knowledge on flowering control from one *Rosaceae* species to the other. An earlier study from Iwata *et al.* (2012) demonstrated that variants from the same flowering gene (*TFL1*) produced naturally through different means in rose (transposon insertion) and woodland strawberry (amino acid deletion) conferred the same CF trait in the two species. Seasonality- and light-responsive quantitative trait loci (QTLs) have been identified in several species including the cultivated strawberry (Perrotte *et al.*, 2016*a*, *b*) and also the less studied raspberry (Jibran *et al.*, 2019). Furthermore, in raspberry, the annual fruiting (AF) trait in which fruiting occurs in the same year as floral initiation, as for the CF trait in strawberry, maps to a genomic region syntenic to that harbouring the CF trait in cultivated strawberry (Jibran *et al.*, 2019).

In this context, the findings from Lu *et al.* (2020) highlighting the role of CO in day neutrality in rose should be readily exploited to extend our understanding of flowering control in other *Rosaceae* species. The recent availability of high-quality genome sequences for major diploid and polyploid *Rosaceae* crop species and of efficient gene editing technologies further open the way to identify genetic variations modulating flowering and to tailor crop varieties with improved flowering characteristics and yield (Gaston *et al.*, 2020).
